# Impaired anti-fibrotic effect of bone marrow-derived mesenchymal stem cell in a mouse model of pulmonary paracoccidioidomycosis

**DOI:** 10.1371/journal.pntd.0006006

**Published:** 2017-10-17

**Authors:** Julián Camilo Arango, Juan David Puerta-Arias, Paula Andrea Pino-Tamayo, Lina María Salazar-Peláez, Mauricio Rojas, Ángel González

**Affiliations:** 1 Medical and Experimental Mycology Group, Corporación para Investigaciones Biológicas (CIB)–Universidad de Antioquia, Medellín, Colombia; 2 School of Microbiology, Universidad de Antioquia, Medellín, Colombia; 3 Department of Microbiology and Immunology, Weill Cornell Medical College, New York, New York, Unites States of America; 4 Basic Sciences Group, Universidad CES, Medellín, Colombia; 5 Dorothy P. & Richard P. Simmons Center for Interstitial Lung Disease, School of Medicine, University of Pittsburgh, Pittsburgh, Pennsylvania, Unites States of America; 6 Basic and Applied Microbiology Research Group (MICROBA), Universidad de Antioquia, Medellín, Colombia; Rutgers University, UNITED STATES

## Abstract

Bone marrow-derived mesenchymal stem cells (BMMSCs) have been consider as a promising therapy in fibrotic diseases. Experimental models suggest that BMMSCs may be used as an alternative therapy to treat chemical- or physical-induced pulmonary fibrosis. We investigated the anti-fibrotic potential of BMMSCs in an experimental model of lung fibrosis by infection with *Paracoccidioides brasiliensis*. BMMSCs were isolated and purified from BALB/c mice using standardized methods. BALB/c male mice were inoculated by intranasal infection of 1.5x10^6^ *P*. *brasiliensis* yeasts. Then, 1x10^6^ BMMSCs were administered intra venous at 8^th^ week post-infection (p.i.). An additional group of mice was treated with itraconazole (ITC) two weeks before BMMSCs administration. Animals were sacrificed at 12^th^ week p.i. Histopathological examination, fibrocytes counts, soluble collagen and fibrosis-related genes expression in lungs were evaluated. Additionally, human fibroblasts were treated with homogenized lung supernatants (HLS) to determine induction of collagen expression. Histological analysis showed an increase of granulomatous inflammatory areas in BMMSCs-treated mice. A significant increase of fibrocytes count, soluble collagen and collagen-3α1, TGF-β3, MMP-8 and MMP-15 genes expression were also observed in those mice. Interestingly, when combined therapy BMMSCs/ITC was used there is a decrease of TIMP-1 and MMP-13 gene expression in infected mice. Finally, human fibroblasts stimulated with HLS from infected and BMMSCs-transplanted mice showed a higher expression of collagen I. In conclusion, our findings indicate that late infusion of BMMSCs into mice infected with *P. brasiliensis* does not have any anti-fibrotic effect; possibly because their interaction with the fungus promotes collagen expression and tissue remodeling.

## Author summary

This is the first study that evaluates the effect of BMMSCs therapy for lung fibrosis induced by the fungal pathogen *Paracoccidioides brasiliensis*, the causative agent of paracoccidioidomycosis, one of the most important systemic endemic mycosis diagnosed in South America and Central America. Our findings showed an impaired anti-fibrotic effect of BMMSCs transplantation. This effect could be triggered by either the chronic inflammatory microenvironment induced by *P*. *brasiliensis* or by a direct interaction between BMMSCs and the fungus, resulting in an exacerbation of the pulmonary fibrosis. In fact, the pro-fibrotic effect exerted by BMMSCs was toned-down by the usage of the antifungal ITC.

## Introduction

Bone marrow-derived mesenchymal stem cells (BMMSCs) are adult stem cells capable of both renew themselves and differentiate in vitro into multiple cell lineages [1]. These cells can also modulate the inflammatory response and induce tissue regeneration through release of cytokines, chemokines, growth factors and genetic material (e.g. miRNAs) [2–6]. Likewise, their microbicidal properties have been already described [7–10]. Cell-based therapies in regenerative medicine using syngeneic or autologous BMMSCs are considered a promising approach, because they do not induce tissue rejection and exert a localized effect than the systemic classical pharmacological strategies [11].

BMMSCs have also been evaluated in animal models of acute lung injury induced by chemicals, such as bleomycin [12, 13] and hydrochloric acid (HCl) [14]. These studies have shown that BMMSCs secrete cytokines, chemokines, growth factors and extracellular matrix proteins, and that can influence the magnitude and quality of the immune response (e.g. modulating the inflammatory response), and promote tissue repair. Likewise, BMMSCs can differentiate into pulmonary stromal cells (e.g. lung fibroblasts and myofibroblasts) [12, 14, 15].

Pulmonary fibrosis (PF) is a process characterized by excessive deposition of collagen and extracellular matrix components that results in a pathological remodeling of the pulmonary architecture. Thus, patients with PF exhibit radiographic, but also functional and clinical alterations in the lung [16]. From the pathological perspective, PF is a dynamic process involving immune system cells and soluble factors including leukotrienes, cytokines (IFNγ, TNFα, IL1, IL4, IL6, IL17), chemokines (CCL2, CCL3, CXCL12), reactive oxygen species (ROS), growth factors [platelet-derived growth factor (PDGF), vascular endothelial growth factor (VEGF), insulin-like growth factor (IGF)], and membrane-bounded and soluble molecules such as prostaglandins, metalloproteinases (and their tissue inhibitors), among others [17]. An imbalance between pro-fibrotic responses and anti-inflammatory and pro-tissue repair agents, results in the differentiation and activation of myofibroblasts which once activated produce abundant amounts of collagen, thus inducing fibrosis of the pulmonary parenchyma [18]. The participation of all the previous components in the PF has been extensively studied in animal models [16, 18, 19].

Pulmonary fibrosis can be induced by microbial agents including the dimorphic fungal pathogen *Paracoccidioides brasiliensis*, the causative agent of paracoccidioidomycosis (PCM), disease that is considered one of the most important endemic systemic mycosis in South America and Central America [20–23]. Brazil, Colombia and Venezuela are the countries with the highest number of cases reported so far, with an estimated of 10 million people infected [20, 21]. The chronic form of PCM is the most frequent clinical presentation (90% of the cases), and it is characterized by a granulomatous inflammatory response with fibrosis development and loss of respiratory function, which is observed in 60% of the patients [22]. Itraconazole (ITC) is the treatment of choice in PCM [23]. Nonetheless, it exerts a fungistatic effect against *P*. *brasiliensis in vivo*, and it does not attenuate the pulmonary alterations induced by the fungal infection, including fibrosis [24, 25]. Animal models of PCM have allowed characterization of the mechanisms involved in the development of pulmonary fibrosis, and evaluate diverse strategies to treat it. Thus, combined therapies including pentoxifylline plus ITC [26], or an anti- neutrophil monoclonal antibody alone or in combination with ITC [27, 28], have showed that such treatment strategies reduced substantially the PF. However, the potential risk for using immunosuppressive drugs or biological agents, mainly in host with other unknown latent infections, should be considered [29].

The objective of this study was to investigate the regenerative effect of BMMSC on PF induced by the fungal pathogen *P*. *brasiliensis* in an *in vivo* experimental model of PCM; nonetheless, our findings indicated that these cells impaired the anti-fibrotic effect and by the contrary, an exacerbated fibrotic process was observed.

## Materials and methods

### BMMSCs isolation, purification and characterization

BMMSCs were obtained from four weeks-old BALB/c mice from the breeding colony maintained at the *Corporación para Investigaciones Biológicas* (CIB, Medellín-Colombia). The BMMSCs isolation and purification protocols were adapted from a protocol previously described by Rojas *et al* [12]. Briefly, mice were anesthetized with a solution of ketamine (80 mg/kg) and Xylazine (8 mg/kg) via intramuscular. Femurs and tibias were removed and bone marrow cells were isolated by flushing with Dulbecco's Modified Eagle Medium (DMEM)-low glucose (GIBCO, Invitrogen Corporation, Carlsbad, CA, USA) containing penicillin/streptomycin1% (vol/vol) (GIBCO). Cells were transferred to cell culture flasks (Eppendorf, Hamburg, Germany) with DMEM-low glucose supplemented with 10% (vol/vol) fetal bovine serum (FBS) (GIBCO) and nonessential amino acids 1% (vol/vol) (GIBCO), followed by incubation at 37°C in 5% CO_2_. Non-adherent cells were removed after 48 hours, and maintained in standard culture media for 7 days.

In order to exclude hematopoietic stem cells and leucocytes, a magnetic bead-based mouse cell depletion kit (Miltenyi Biotec, Bergisch Gladbach, Germany) containing anti-CD45, anti-CD11b, anti-CD5, anti-Gr1 (Ly-6/C), and anti-Ter 119 monoclonal antibodies was used. The BMMSC surface markers expression profile was determined by flow cytometry. The following antibodies were used: isothiocyanate (FITC) anti-CD45 (BD Pharmingen, San Diego, CA, USA), phycoerythrin (PE)-Cy5-anti-CD44, allophycocyanin (APC) anti-CD105, PE-Cy7-anti-CD106, APC-anti-TER-119, Pacific blue-anti-SCA-1, and PE-anti-CD73 (Biolegend, San Diego, CA, USA). Cells were analyzed using a FACS Canto II system (BD Biosciences, San Jose, CA, USA) and FlowJo V10 software (FlowJo, LLC, Data Analysis software, Ashland, OR, USA). In addition, a differentiation assay to demonstrated the BMMSCs plasticity (differentiation to chondrogenic, adipogenic and osteogenic lineages) was performed using a differentiation commercial kit, and following the manufacturer’s instructions [StemPro (Waltham, MA, USA)]. Finally, the purified cells were kept in standard culture media until the day of transplant.

### Ethical statement

This study was carried out following the Colombian (Law 84/1989, Resolution No. 8430/1993), European Union, and Canadian Council on Animal Care regulations. The protocol was approved by the Institutional Ethics Committee of the CIB (Acta No.95).

### Mouse model of chronic pulmonary paracoccidioidomycosis

A highly virulent strain of *P*. *brasiliensis* (Pb18) was used in order to develop the experimental pulmonary fibrosis model as described previously [28]. Briefly, BALB/c male mice (8 weeks old) were intranasally infected with 1.5 x 10^6^ *P*. *brasiliensis* yeast cells contained in 60 μl of phosphate-buffered saline (PBS). The total inoculum was split into two equal doses, which were instilled within a 5–10 minutes (min) period. Non-infected (control) mice were inoculated with 60 μl of PBS.

### BMMSCs transplant

Infected and non-infected mice were intravenously injected with 1x10^6^ BMMSCs at 8^th^ week post-challenged given in a single dose. Six week post-inoculation, an additional group of infected animals was treated with 100 μl of Itraconazole (ITC) oral solution (Sporanox, Janssen-Cilag S.A., Mexico) administered at a dose of 1mg/day in order to achieve serum levels equal to 1 μg/mL. The above treatment was administrated daily and uninterruptedly for 6 weeks by gavage. All animals included in the various experimental groups were sacrificed at week 12^th^ p.i. and their lungs harvest for further studies.

### Fibrocytes count by flow cytometry

Lungs of mice were removed, homogenized and sequentially filtered through 70 and 40μm sterile cell strainers (Thermo Fisher Scientific Inc, Waltham, MA, USA) in RPMI cell culture medium plus 1% (vol/vol) FBS (Sigma-Aldrich, Saint Louis, MO, USA). Cells suspension were centrifuged at 500 G, 10°C for 10 min, and red blood cells were lysed using the ACK Lysing Buffer (GIBCO). Viability of the cells was determined by trypan blue exclusion test with samples being used if they were 95% of viable. Cells were resuspended in RPMI plus 10% FBS and counted using a hemocytometer. Fc receptors were blocked using a purified rat anti-mouse CD16/CD32 (BD Pharmigen, San Diego, CA, USA). Then, cells were treated with Cytofix/Cytoperm and Perm/Wash solution (BD Pharmigen, San Diego, CA, USA) [28]. Fibrocytes were determined using FITC anti-collagen I (Rocklad inc Limerick USA), PE anti-CD45 (Biolegend San Diego USA), and APC anti-CD34 (BD Pharmingen, San Diego USA). Anti-mouse IgG-FITC (Rocklad), anti-mouse IgG2aκ-PE (Biolegend) and anti-mouse IgG1κ-APC (BD) were used as isotype controls. The stained cell suspensions were fixed with FACS buffer/1% (vol/vol) PFA (Carlo Erba, Barcelona, Spain). Assays were performed using a FACS Canto II system (BD Biosciences, San Jose, CA, USA), while information analysis were done using FlowJo V10 (FlowJo, LLC, Data Analysis software, Ashland, OR, USA). Fibrocyte population was analyzed as follows: (a) cell events in region 1 (R1) were gated by forward scatter versus side scatter areas; (b) CD45+ events were gated from R1 by side scatter area versus CD45 staining to establish the R2 region, from which (c) cell events were gated to determine fibrocytes by collagen 1+ (intracellular) and CD34+ (surface). The number of fibrocytes was determined by multiplying the percentage of the gated population by the total number of leukocytes (CD45+ population).

### Histopathological analysis

Lungs were processed and analyzed as described by Puerta-Arias *et al* [28]. Briefly, lungs were perfused with 1X PBS to wash out red blood cells. Tissue fixation was completed in a 4% buffered formalin solution. Then, fixed tissues were embedded in paraffin and sections stained with Masson trichrome, and examined using a Nikon Eclipse Ci-L microscope—Nikon DS-Fi2 digital camera. A morphometric analysis was performed using NIS Elements 4.30.02 Laboratory Image Software (Nikon Instruments Inc., Melville, USA). The percentage of occupied area by the inflammatory response was calculated by dividing the total inflamed area, which includes cellular infiltrates and granulomatous lesions by the total area of the lung.

### Soluble collagen determination

Homogenized lung suspensions were treated with acid neutralizing reagent (0.5M acetic acid, 0.1 mg/ml pepsin) (Sigma-Aldrich, Saint Louis, MO, USA). Then, colorimetric detection of soluble collagen content was performed according to the manufacturer's protocol of a sircol collagen assay kit (Biocolor, Northern Ireland, U.K.). A calibration curve was constructed using bovine collagen-I in the range of 1–10 μg.

### Determination of collagen expression by human fibroblasts stimulated with homogenized lung supernatants from experimental animals

Human lung fibroblasts were obtained from Rojas’ Lab repository, collected under an established protocol from the University of Pittsburgh Center for Organ Research Involving Decedents (CORID). Cultures of human fibroblasts (2x10^4^ cells/200uL, pass 4) were treated with soluble lungs supernatants (protein concentration 10ug/mL) from all experimental groups, for 24h at 37°C. Then, fibroblast activity was determined by measuring the expression of collagen type-I gen using reverse transcriptase real-time-PCR (RT-qPCR) assays, as previously described [29]. As controls, we used PBS and TGF-β [(5ng/ml final concentration) Peprotech Rocky Hill, United States].

### Real time PCR analysis

All real time PCR assays were performed as previously described [28]. Briefly, RNA was obtained from lungs of mice using Trizol (Invitrogen, Carlsbad, CA, USA). Samples were treated with DNase I (Thermo Fisher Scientific Inc, Waltham, MA, USA), and cDNA was synthesized using 500ng of total RNA using cDNA synthesis kit for RT-qPCR according to the manufacturer’s instructions (Thermo Fisher Scientific Inc, Waltham, MA, USA). Real-time PCR was done using Maxima EVAGreen/Fluorescein qPCR Master Mix according to the manufacturer’s instructions (Applied Biological Materials ABM Inc, Richmond, Canada). The CFX96 Real-Time PCR Detection System (Bio-Rad, Headquarters Hercules, California, USA) was employed to measure gene expression levels. Melting curve analysis was performed after the amplification phase of real time PCR assays to eliminate the possibility of non-specific amplification or primer-dimer formation. Validation of housekeeping genes for normalization mRNA expression was performed before gene expression analysis. Expression of fibrosis-related genes encoding for collagen, transforming growth factor beta (TGF-β), matrix metalloproteinases (MMP) and tissue inhibitor of metalloproteinases (TIMP) were evaluated. Fold changes in the target gene mRNA expression were quantified relative to glycer-aldehyde-3-phosphate dehydrogenase (GAPDH the housekeeping gene previously defined) [28]. Each experiment was repeated twice using 5 mice per each one of the groups with gene expression analysis being conducted by triplicate.

### Statistical analysis

Data analysis was performed using Graph Pad Prism software version 7 (GraphPad Software, Inc., La Jolla, CA, USA). Normality for all values was calculated by the Shapiro-Wilk test and when comparisons between three or more groups were required, the ANOVA test was employed. On the other hand, comparisons between two specific groups were determined by Student-t test. Mean and standard error of the mean (SEM) were calculated for all analyses. We considerate *P<0*.*05* values as significant.

## Results

### BMMSCs therapy induced an increase in pulmonary inflammation and fibrosis in the experimental model of paracoccidioidomycosis

**Fig 1:**
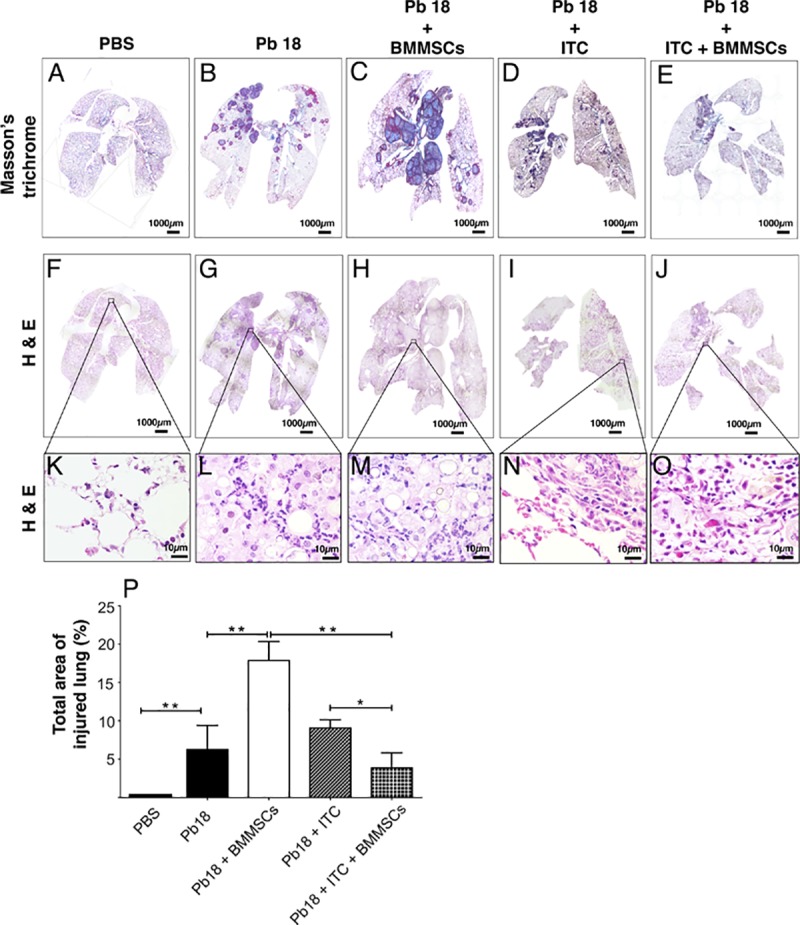
Bone marrow mesenchymal stem cells (BMMSCs) induced an increase in pulmonary inflammation and fibrosis in lungs of mice infected with *P*. *brasiliensis*. Microphotographs A, F, K represent lungs from uninfected mice; B, G, L corresponded to mice infected with *P*. *brasiliensis* (Pb18); C, H, M were taken from infected and BMMSCs-treated animals at 8^th^ week post-challenge; infected and Itraconazole (ITC) treated-mice at 6^th^ week p.i. in D, I, N and samples from infected mice treated with ITC at 6^th^ week p.i. and transplanted with BM-MSCs at 8^th^ week post-challenge as shown in E, J, O. Lungs were fixed in formalin, embedded in paraffin, cut and stained with Masson’s trichrome stain to determine injured and fibrotic lungs areas (A-E) and H&E was used to evaluate the inflammatory response *in situ* (F-O). Lungs stained sections were scanned using a Nikon Eclipse Ci-L microscope—Nikon DS-Fi2 digital camera and analyzed using NIS Elements 4.30.02 Laboratory Image Software. Magnification 4X (A-J) and 100X (K-O). The percentage of injured area was calculated by dividing the total inflamed area, which includes cellular infiltrates and inflammatory lesions (granulomas) by the total area of the lung (P). Data shown represent mean and SEM (n = 4–5 mice/group, representative of two independent experiments). *, *P*<0.05 comparing *P*. *brasiliensis*-infected and BM-MSCs- and ITC-treated mice versus *P*. *brasiliensis*-infected and ITC-treated mice; and **, *P*<0.001 comparing uninfected versus *P*. *brasiliensis*-infected mice, or *P*. *brasiliensis*-infected and BM-MSCs-treated mice with either *P*. *brasiliensis*-infected control mice or *P*. *brasiliensis*-infected and BM-MSCs- and ITC-treated mice.

We determined the granulomatous inflammatory areas through histopathological analysis in lung of experimental mice. We observed that lungs of *P*. *brasiliensis* infected-mice developed a granulomatous inflammatory response with collagen fibers surrounding granulomas (Fig 1A). Interestingly, administration of BMMSCs in infected mice showed an exacerbation of the inflammatory process, with a higher granulomatous inflammation and fibrosis areas with loss of parenchyma (Fig 1B). In contrast, the infected animals treated with ITC alone showed inflammatory and fibrotic responses similar to those infected and non-transplanted mice (Fig 1C). Moreover, the combined administration of BMMSCs/ITC in *P*. *brasiliensis*-infected mice considerably decreased the inflammatory response and fibrosis (Fig 1D), in comparison with those infected animals that only received cell-based therapy. A morphometric analysis revealed that occupied area by granulomatous inflammation in infected and transplanted mice was twice higher when compared with infected non-treated mice (p<0.001), or three time that infected and BMMSCs/ITC-treated animals (p<0.001) (Fig 1E). There was statistically significant difference in the average of occupied area by granulomas between infected and ITC-treated mice and those that received combined therapy (p<0.005).

### BMMSCs administration increases the number of fibrocytes in lungs

**Fig 2:**
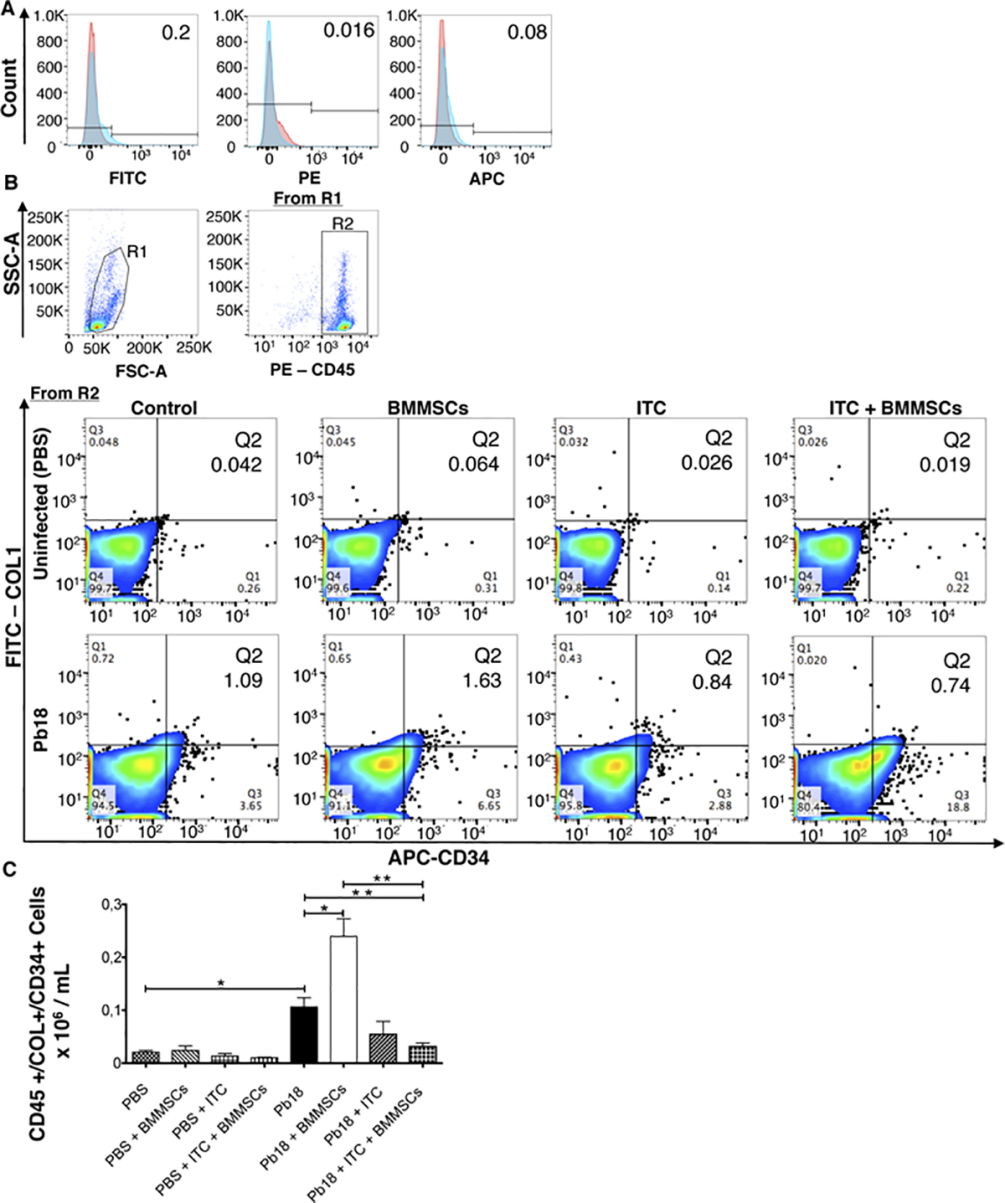
BMMSCs therapy increases fibrocytes in lungs of *P*. *brasiliensis* infected mice. BALB/c mice were infected with *P*. *brasiliensis* yeast (Pb18) and treated with BM-MSC and/or ITC as described in Materials and Methods section. Fibrocytes were assessed by flow cytometry as follows: A) correspond to representative plots of unstained cells (red) and of those treated with the isotype controls (blue). B) correspond to representative plots of gating strategy to determine fibrocytes; R1: represents forward scatter areas (FSC-A) versus side scatter areas (SSC-A) gated cell events; R2: CD45+ pictures cell events gated from R1 by SSC-A versus PE-CD45; finally, fibrocytes were gated from R2 employing FITC-collagen 1+ versus APC-CD34+ (Q2). C) corresponds to absolute number of fibrocytes (CD45+/COL1+/CD34+). Data shown represent mean and SEM of 4–5 mice/group representing two independent experiments. *, *P*< 0.05, when comparing infected untreated mice with uninfected mice, or comparing *P*. *brasiliensis*-infected untreated mice with *P*. *brasiliensis*-infected and BM-MSCs-treated mice; **, *P*<0.001 comparing *P*. *brasiliensis*-infected and BM-MSCs- and ITC-treated mice with either *P*. *brasiliensis*-infected untreated mice or with *P*. *brasiliensis*-infected and BM-MSCs-treated mice.

Fibrocytes are bone marrow-derived fibroblast progenitor cells that have been implicated in tissue remodeling or repairing process, including the development of fibrosis. Following flow cytometry analysis we found a significantly increased number of fibrocytes (CD45^+^/CD34^+^/Collagen1^+^) in lungs from infected mice (p<0.005) (Fig 2) relative to PBS instilled controls. Moreover, infected and BMMSCs-treated animals showed almost twice the number of fibrocytes when compared with infected non-treated mice (p<0.005) (Fig 2). Interestingly, ITC treatment, in combination with BMMSCs, reduced the fibrocytes counts, versus *P*. *brasiliensis* infected mice (p<0.001) or infected BMMSCs-treated animals (p<0.001) (Fig 2).

### BMMSC therapy increases lung soluble collagen content

**Fig 3:**
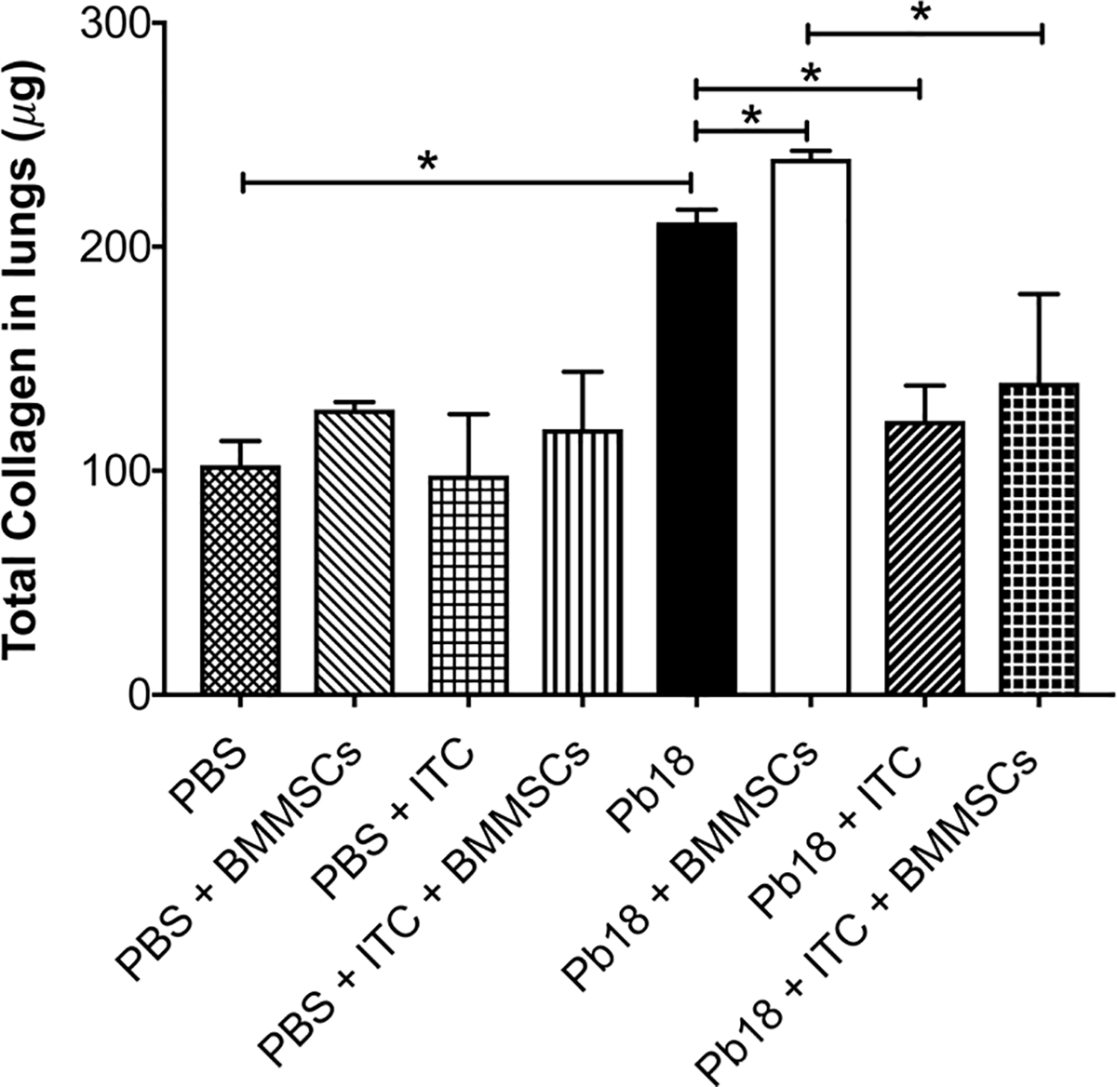
BMMSCs administration increases the total collagen levels in lungs of mice infected with *P*. *brasiliensis*. Total collagen was performed using Sircol Dye reagent as described in Materials and Methods section. Data shown represent mean and SEM (n = 4–5 mice/group; representative of two independent experiments). *, *P*< 0.05, comparing infected untreated mice with uninfected mice; or comparing *P*. *brasiliensis*-infected untreated mice with either *P*. *brasiliensis*-infected and BM-MSCs-treated mice or *P*. *brasiliensis*-infected and ITC-treated mice; or comparing *P*. *brasiliensis*-infected and BM-MSCs-treated mice with *P*. *brasiliensis*-infected and BM-MSCs and ITC-treated mice.

Collagen is considered the most important extracellular matrix protein involved in fibrosis. Accordingly, we determined the effect of BMMSCs therapy on lung soluble collagen content. Significantly increased levels of collagen in lungs from mice infected with *P*. *brasiliensis* were observed when compared with PBS instilled animals (p<0.005) (Fig 3). Infected and BMMSCs-treated mice exhibited an increased significantly in collagen content relative to infected non-treated animals (p<0.001) (Fig 3). Remarkably, ITC treatment reduced the amount of soluble collagen in the lungs from both, *P*. *brasiliensis* infected-mice (p<0.005), or those with BMMSCs transplantation (p<0.005) (Fig 3).

### Human fibroblasts stimulated with homogenized lung supernatants from experimental animals show increased collagen gene expression

**Fig 4:**
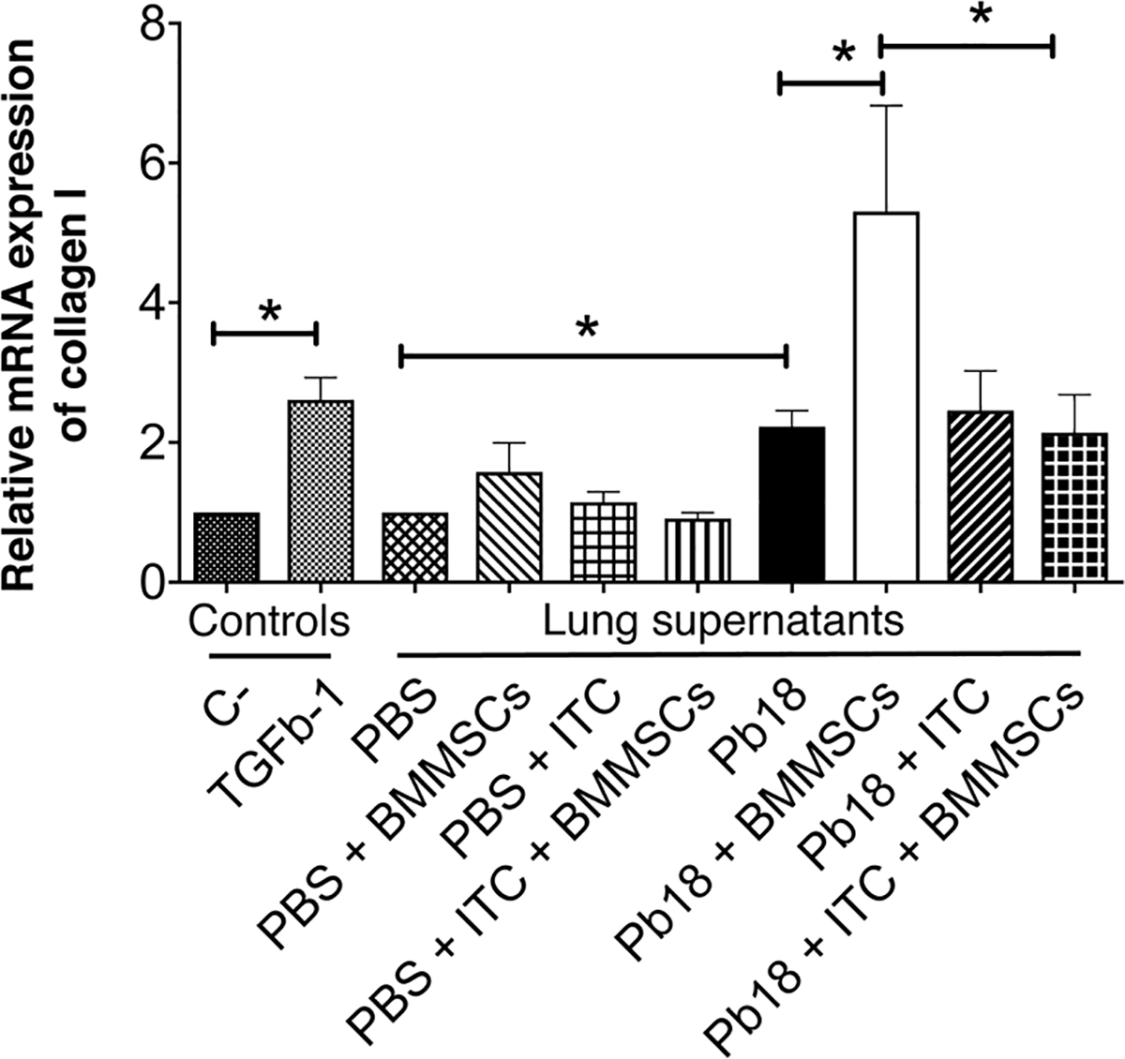
Lung supernatants from mice infected with *P*. *brasiliensis* and treated with BMMSCs induces high expression of collagen I gen by human fibroblasts. Lung human fibroblasts were stimulated with soluble lung supernatants from *P. brasiliensis*-infected mice treated with either BMMSCs and/or ITC, for 24h at 37°C. Fibroblast activation was determined by measuring the expression of collagen I gen by real-time PCR as described in Materials and Methods section. Data shown represent mean and SEM (n = 4–5 mice/group; representative of two independent experiments). *, *P*< 0.05, comparing TGFβ-1-stimulated fibroblast with unstimulated cells; or comparing infected untreated mice with either uninfected mice or *P*. *brasiliensis*-infected and BM-MSCs-treated mice; or comparing *P*. *brasiliensis*-infected and BM-MSCs-treated mice with *P*. *brasiliensis*-infected and BM-MSCs and ITC-treated mice.

To assessment the capability of lung homogenized to activate human lung fibroblasts, we performed *in vitro* assays stimulating fibroblasts with lung supernatants from experimental animals. We observed that lung supernatants from infected and BMMSCs-treated mice induced a higher expression of the gene encoding for collagen type-I in human fibroblast, in comparison with the respective homogenized lung supernatants from the infected non-treated mice (p<0.005) (Fig 4). The homogenized lung supernatants from mice infected with *P*. *brasiliensis* and treated with ITC, alone or in combination with BMMSCs transplantation, in human fibroblast also induced a collagen gene expression similar to that found in human fibroblast stimulated with lung homogenates from infected non-treated-mice (Fig 4).

### BMMSCs administration alters the expression of genes related with fibrosis development

**Fig 5:**
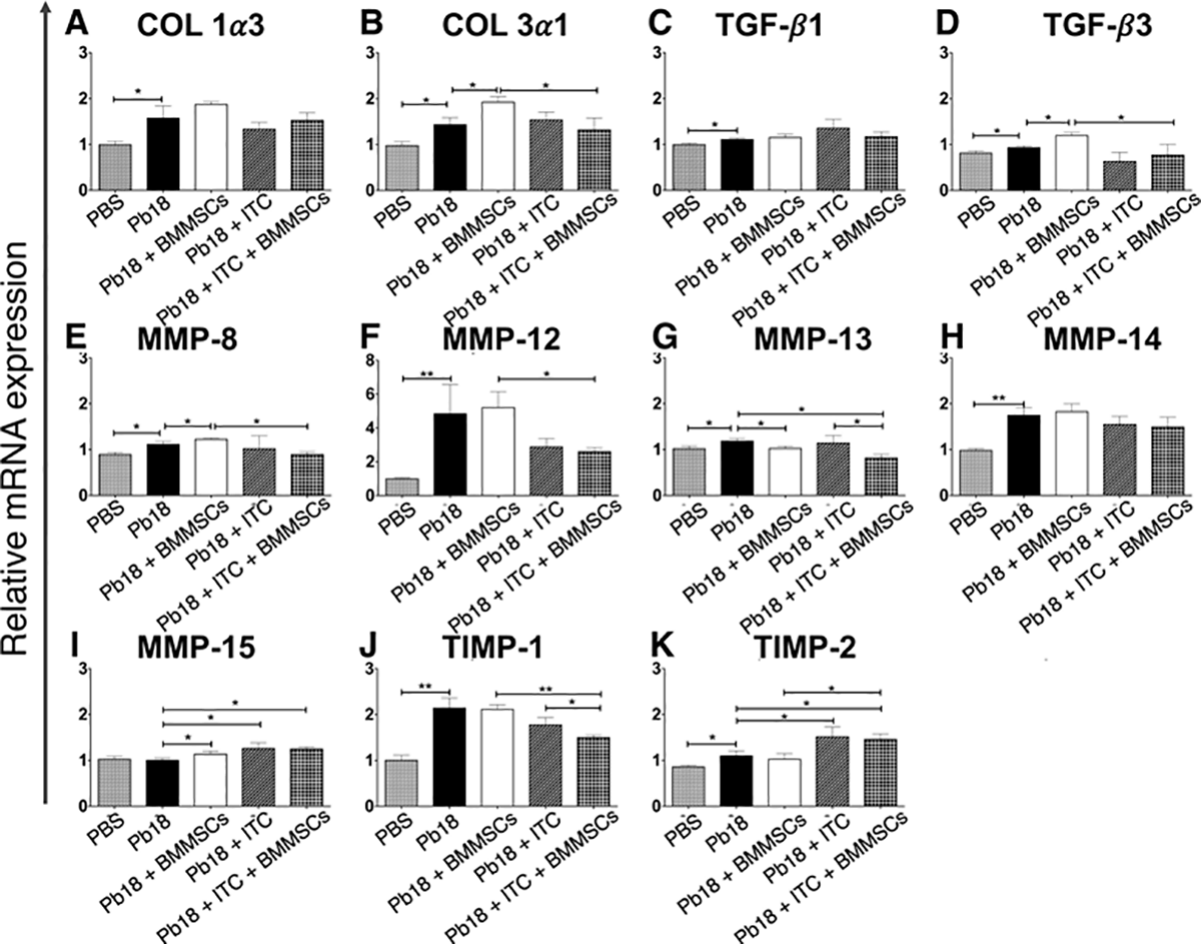
BMMSCs therapy alters the expression of genes related with the fibrosis process in lungs of mice infected with *P*. *brasiliensis*. Relative quantification of the mRNA expression of fibrosis related genes were performed in lungs of mice. A) COL 1α3; B) COL 3α1; C) TGF-β1; D) TGF-β3; E) MMP-8; F) MMP-12; G) MMP-13; H) MMP-14; I) MMP-15; J) TIMP-1; and K) TIMP-2. Data shown represent mean and SEM (n = 4–5 mice/group; representative of two independent experiment). *, *P*< 0.05, **, *P*<0.001 comparing infected untreated mice with uninfected mice, or comparing *P*. *brasiliensis*-infected untreated mice with *P*. *brasiliensis*-infected and BM-MSCs-treated mice; or comparing *P*. *brasiliensis*-infected untreated mice with *P*. *brasiliensis*-infected and ITC-treated mice; or comparing *P*. *brasiliensis*-infected and BM-MSCs-treated mice with *P*. *brasiliensis*-infected and ITC-treated mice; or comparing *P*. *brasiliensis*-infected and BM-MSCs-treated mice with *P*. *brasiliensis*-infected and BM-MSCs and ITC-treated mice, respectively. COL, collagen; TGF-β, transforming growth factor beta; MMP, matrix metalloproteinases; TIMP, tissue inhibitor of metalloproteinases.

Our next step was to determine if the BMMSCs administration affects the related-fibrotic response genes expression in lungs from mice infected with *P*. *brasiliensis*. We observed that *P*. *brasiliensis* infected-mice showed a higher expression of almost all genes evaluated (Col1α3, Col3α1, TGF-β1, TGF-β3, MMP-8, MMP-12, MMP-13, MMP14, TIMP-1 and TIMP-2) when compared with uninfected mice. Moreover, after BMMSCs treatment, a significantly higher expression of Col3α1, TGF-β3, MMP-8, and MMP-15 was observed in comparison with those *P*. *brasiliensis* infected-mice, while a reduction on MMP-13 gene expression was also observed (Fig 5). Infected mice that received the ITC treatment showed a slight but higher expression of the MMP-15 and TIMP-2 genes relative to infected non-treated animals. Remarkably, the combined therapy BMMSCs/ITC induced a synergistic reduction of Col3α1, TGF-β-3, MMP-8, MMP-12, and TIMP-1, as well as an increase of TIMP-2 gene expression, when compared to infected mice that received cell transplantation (Fig 5).

## Discussion

Pulmonary fibrosis (PF) is a serious disease triggered by chemical, physical, or infectious agents, but also it could be an idiopathic or cryptogenic process [16, 18]. The fungus *P*. *brasiliensis* is the etiological agent of paracoccidioidomycosis (PCM), a disease endemic in Latin America. In the chronic form of the disease, more than 60% of patients develop fibrotic sequelae compromising lung parenchyma, even after the completion of treatment [22]. In fact, the current therapeutic strategy for PCM is based on azole compounds as itraconazole (ITC), an antimycotic that reduces fungal load but not PF development [20]. Therefore, in recent years, we had been focused to evaluate alternative therapies in an attempt to reduce the fibrotic response in this disease [22, 28]. In the present study, we investigated for the first time the effect of a BMMSCs-based cellular therapy on PF in a murine model of PCM. Nonetheless, contrary to other reports showing a beneficial effect of BMMSCs transplants in PF due to chemical or physical agents [30–32], we observed that this type of cellular therapy exacerbated the fibrotic response.

BMMSCs are considered as promising in the development of cellular therapies due to their capacity to regenerate tissue, as well as for their immunomodulatory properties, which include the release of paracrine or endocrine signaling molecules [5, 32, 33]. Moreover, different studies have shown that BMMSCs are able to sense the microenvironment and respond to both physical (i.e. mechanical) and chemical stimulus [34, 35]. Namely, it has been recognized that extracellular matrix (ECM) influences stem cell lineage commitment [35]. As an example, Li *et al*. [34, 35] described that fibronectin, a glycoprotein that connects integrins in cell surface with collagen fibers in the ECM, playing a role as mechanotransducer signal that regulate human mesenchymal stem cells (hMSC) differentiation [34]. Our group have previously shown that lungs of mice infected with *P*. *brasiliensis* exhibit an increased expression and re-arrangement of ECM components (e.g. collagen, fibronectin, laminin, proteoglycans), even after two days post-infection, but fully established after 4^th^ week post-infection [36, 37]. Accordingly, that early deposition could favors the BMMSCs differentiation to fibrocytes, who in turn can proliferate and undergo phenotypic conversion to fibroblast or myofibroblast, the cellular culprits of fibrosis [38]. In fact, after BMMSCs administration, we observed a rise of fibrocytes counts and soluble collagen content in lungs from infected-mice.

In addition to their ability to interact with ECM proteins, BMMSCs may also recognize microbial compounds through pattern recognition receptors (PRRs), interactions that may induce a pro-inflammatory response [35, 38]. Bernardo *et al* [39], just as Waterman *et al* [40], have demonstrated that the activation of Toll-like receptors (TLRs) in mesenchymal stem cells promotes their polarization into a pro-inflammatory phenotype, named *MSC1*, which can fuel inflammation and subsequent fibrosis [39, 40]. In this sense, it has been reported that the interaction between *P*. *brasiliensis* and TLR4 lead to a severe fungal infection, associated with an enhanced exacerbated proinflammatory response [41]. All these reports support both the fungal proliferation and tissue damage observed after BMMSCs administration, and suggest an immunoregulatory role of these stem cells; thus, the deleterious effect observed maybe triggered by the interaction of BMMSCs with either *P*. *brasiliensis* compounds, extracellular matrix and the inflammatory microenvironment developed during the chronic course of PCM. However, more studies are needed to evaluate the interaction between BMMCSs and this fungal pathogen, as well as its implications not only for the immune response, but also for tissue repair.

Besides that, it is worth to highlight that myofibroblasts might be derived from other cellular sources beyond bone marrow fibrocytes. In fact, these collagen-producing cells, and main effectors of fibrosis, may arise from resident fibroblast, or as a result of epithelial/endothelia mesenchymal transitions [42]. In this study, the administration of BMMSCs was associated with an increase in the number of fibrocytes. Although, the origin of these cells could not be confirmed, we may suppose that they could come from bone marrow or pericytes, which subsequently differentiate into fibroblasts and then into myofibroblasts collagen-producer cells, thus contributing to the increase of PF in those *P*. *brasiliensis* infected- and BMMSCs treated-mice. Accordingly, we evaluated the collagen I gene expression in human fibroblast stimulated with homogenized lung supernatants from *P*. *brasiliensis* infected mice. We found that supernatants from infected and BMMSCs-transplanted mice induced a higher production of type I collagen transcript in human fibroblast, in comparison with those cells stimulated with infected non-treated animals. These results clearly indicate the presence of molecules from the lung microenvironment able to stimulate the collagen production in human fibroblast. Among the major stimuli to activate fibroblasts, IL6, TGFβ, IL13 and FGF are found, and once activated, these cells differentiate into myofibroblasts or could produce pro-fibrotic molecules such as IL1, VEGF, Insulin-like growth factor 2 (IGFII), Insulin-like growth factor-binding protein (IGFBP), IL6 and IL33 [42]. In fact, we observed an increased expression of Col 3α1 and TGFβ-3 genes in lungs of *P*. *brasiliensis*–infected mice treated with BMMSCs. In concurrence with these reports, more recently we have observed a significant increase of IL-1α, IL-1β, IL-6, TNF-α and IL17 levels in homogenized lung supernatants from mice infected with *P*. *brasiliensis* [28]. All these cytokines have also been implicated in the pathogenesis of PF. Nonetheless, a direct activation of fibroblast by *P*. *brasiliensis* compounds could not be ruled out.

Matrix metalloproteinases (MMPs) are a family of zinc- and calcium-dependent endopeptidases (around 25 members are know so far) that are either secreted or membrane-bound enzymes [43]. MMPs have long been considered to be essentials for ECM remodeling, which is critical in embryonic development and tissue homeostasis, including inflammatory response and tissue repair [43]. In this context, as stated by Pardo et al, MMPs not only degrade ECM components, but also release, cleave and active a wide range of growth factors, cytokines, chemokines and cell surface receptors affecting numerous cell functions (e.g. adhesion, proliferation, differentiation, migration, cell death) [44]. Thus, MMPs and their tissue inhibitors (TIMPs) play a central role in the extracellular pathways of ECM degradation and, therefore, in fibrosis development or resolution [44]. Namely, MMP8, a collagenase, can also cleave the chemokines CXCL8 and LIX, resulting in enhanced chemoattractant activities, which could be associated with fibrosis development [43, 44]. These results show that after BMMSC administration there was not only an increase in the expression MMP8 gene but also elevated neutrophils counts, findings noticed before in association with development of fibrosis as observed in our PCM model previously reported [28]. BMMSCs transplantation also induced a decrease in gene coding for MMP-13, another collagenase, but not changes in the expression of TIMP-1 and TIMP2 genes were observed. Meanwhile, the ITC/BMMSC transplantation therapy decreased synergistically the expression of TIMP-1 and MMP-13. Over expression of TIMP-1 is associated whit liver fibrosis [45], while MMP-13 cleaves and inactivates CCL2, CCL7 and CXCL12 leading to reduction in chemotaxis, as well as to a decrease in the fibrosis process [43, 45]. However, our data interpretation relative to MMPs or TIMPs gene expression is limited, as the current knowledge concerning pathological tissue repair in PMC is scarce. In addition, although most of the fibrosis-related genes analyzed showed small fold-changes with statistical significant, a possible meaningful biological effect cannot be ruled-out.

ITC is the antifungal treatment of choice in PCM. Of note, additionally to its antifungal effect, it has been recently documented that this antifungal medication also exhibits immunomodulatory properties [46]. Moreover, in a previous work, we found that ITC reduces the expression of certain genes encoding for pro-inflammatory cytokines (IFN-γ, IL-6, IL-17, TGF-β1, TNF-α), transcriptional factors (T-bet, GATA-3, Spi-1, RoRc, Ahr, FoxP3), and fibrosis development (MMP-1, MMP-8, MMP-13, and Col3α1). Additionally, it also diminished the number of inflammatory cells—including neutrophils—in the lungs of mice infected with *P*. *brasiliensis* [27]. In the present study, it was observed that the ITC regulates the expression of the MMP-15 and TIMP-2 genes that have been recognized as inducers of pulmonary fibrosis [27].

### Conclusions

Overall, our results demonstrated an exacerbating effect of the BMMSCs therapy on pulmonary fibrosis induced by *P*. *brasiliensis* infection. We hypothesized that this outcome could be triggered by either the interaction with *P*. *brasiliensis* compounds or by the inflammatory microenvironment induced by this process. Nonetheless, the combined therapy ITC/BMMSCs showed promising results since synergistically it reduced TIMP-1 and MMP-13. Thus, the use of BMMSC under different conditions or combined with other treatments (e.g. ITC) opens the possibility to new therapeutic approaches for this type of fibrosis resulting from an infectious disease.

PCM is considered a neglected tropical disease mostly affecting low income individuals who live in underdeveloped Latin American rural regions where the technology and the resources needed to administer the immunotherapeutic measures here suggested would probably not be available. Nonetheless, the implementation of cellular therapies is progressing and the prospects are to arrive in a few years to the administration of autologous bone marrow or stem cells obtained from adipose tissues even in these regions.
